# Transarterial chemoembolization with medium-sized doxorubicin-eluting Callisphere is safe and effective for patients with hepatocellular carcinoma

**DOI:** 10.1038/s41598-020-61209-6

**Published:** 2020-03-10

**Authors:** Cao-ye Wang, Jin-guo Xia, Zheng-qiang Yang, Wei-zhong Zhou, Wen-hua Chen, Chun-jian Qi, Jian-ping Gu, Qi Wang

**Affiliations:** 1 Department of Interventional Radiology, The Affiliated Changzhou No.2 People’s Hospital of Nanjing Medical University, Changzhou, China; 2 Department of Interventional Radiology, First People’s Hospital of Changzhou (Affiliated Hospital of Soochow University), Changzhou, China; 3 Hepatobiliary Center, The First Affiliated Hospital with Nanjing Medical University, Nanjing, China; 4 Medical Research Center, The Affiliated Changzhou No.2 People’s Hospital of Nanjing Medical University, Changzhou, China; 5 Department of Interventional Radiology, Nanjing First Hospital (Affiliated Hospital of Nanjing Medical University), Nanjing, China

**Keywords:** Biomarkers, Biomarkers, Energy science and technology, Energy science and technology

## Abstract

The study aimed to compare the tumor response to and complications of doxorubicin-eluting CalliSphere bead-transarterial chemoembolization (DEB-TACE) using small- and medium-sized beads in patients with hepatocellular carcinoma (HCC) who underwent multiple rounds of oncology therapies. Sixty patients with intermediate stage HCC who had previously received multiple oncology therapies underwent DEB-TACE with CalliSpheres of 100–300 μm (small bead group, n = 34) or 300–500 μm (medium bead group, n = 26) in diameter between October 2016 and December 2018. Adverse events and the response rate of the index tumor based on the modified Response Evaluation Criteria in Solid Tumors at 3 months post-TACE were compared between the groups. The rates of complete response, partial response, stable disease, and progressive disease were 35.4%, 29.4%, 17.6%, and 17.6%, respectively, for the small bead group and 33.1%, 23.1%, 20.8%, and 23.0%, respectively, for the medium bead group, showing no significant between-group differences (P > 0.05). Common Terminology Criteria for Adverse Events version 4.0 grade 3/4 adverse events were reported in 8 patients in the small bead group and in no patients in the medium bead group, showing a significant group difference (P < 0.01). Major complications included 8 events of ischemic hepatitis, 2 of biloma, and 2 of severe liver abscess. DEB-TACE using CalliSpheres of 300–500 μm was associated with a comparable rate of tumor response but lower rate of complications compared with that using CalliSpheres of 100–300 μm for HCC treatment in patients who had already undergone multiple rounds of oncology therapies.

## Introduction

Transarterial chemoembolization (TACE) is the current standard of care for patients with intermediate-stage hepatocellular carcinoma (HCC)^1^. In conventional TACE (C-TACE), lipiodol and gelatin sponge particles are commonly used as the embolic agents simultaneously applied with chemotherapeutic drugs that are selectively delivered to the arteries feeding the tumor^2^. C-TACE is a heterogeneous technique with no standardized approach, likely due to the difficulty of obtaining reproducible and stable lipiodol/drug emulsions with a standard droplet size and the lack of consensus on which embolic agent should be used. Additionally, a critical challenge is that the time elapsed between chemotherapy injection and embolic agent placement allows the clearance of a large proportion of drug into the systemic circulation^3^.

Recently, drug-eluting beads (DEBs) have emerged as a novel drug delivery agent for TACE^4^, as they offer the capability of carrying up to a double dose of doxorubicin and overcome problems frequently encountered by C-TACE, including uncontrolled release of chemotherapeutic agents into the systemic circulation^5^. DEB-TACE provides higher levels of consistency and repeatability that offer the opportunity to implement a more standardized approach to treat HCC^6^. DEBs are capable of slowly releasing the loaded chemotherapeutic agent upon injection and increasing the intensity and duration of ischemia while enhancing the drug delivery to the target tumor. Although the time to local progression was not found to differ significantly between cases treated with DEB-TACE and C-TACE, DEB-TACE demonstrated less post-procedural toxicity^7^ and less frequent post-embolic syndrome as compared with C-TACE^8^.

A recent meta-analysis, including data from 1990 to 2015, concluded that DEB-TACE achieved a higher complete response rate and higher overall survival rate than C-TACE in patients with HCC. Moreover, DEB-TACE was found to be safe and associated with fewer adverse events than C-TACE in the treatment of HCC^9^. Recently, another meta-analysis of 30 studies were analyzed found that patients who underwent DEB-TACE had a higher complete response rate, disease control rate, and 3-year survival rate than patients who underwent C-TACE; no significant difference regarding safety was seen for patients treated with C-TACE and DEB-TACE^10^.

As drug-loadable embolic microspheres, the first-generation DEBs had a particle diameter ranging from 500–900 µm. Continuous efforts have been directed toward reducing the particle size of DEBs based on the hypothesis that microspheres of smaller size can penetrate more deeply into the arterioles of tumor tissues. Theoretically, small-size microspheres allow for more homogeneous intratumoral drug distribution and more distal vascular penetration^11^. This theory was supported by a recent study in patients with liver malignancies in whom DEBs with small diameters achieved improved the radiological response in terms of extensive intratumoral necrosis^12^. DEB-TACE with 70–150 µm particles demonstrated an improved 1-month objective tumor response compared to that with 100–300 µm particles in patients with HCC, while having a similar safety profile^13^.

It should be noted that the findings of these studies are specific to patients with treatment-naïve HCC. It remains unclear whether these observations can be extended to patients who have undergone several rounds of first-line therapeutics. Therefore, it is of clinical importance to evaluate the efficacy and safety of DEB-TACE in patients who have received prior treatment for HCC.

CalliSpheres® Beads are the first DEB product available for HCC treatment in China^14^. The aim of this study was to assess and compare the safety and clinical and radiological responses achieved by DEB-TACE using CalliSpheres with diameters of 100–300 µm 300–500 µm in patients with intermediate stage HCC who had received multiple oncological treatments.

## Materials and Methods

### Study population

In this retrospective study, medical records of selected patients were reviewed. This study was approved by the Ethics Committee of Nanjing Medical University. All procedures performed in studies involving human participants were in accordance with the ethical standards of the institutional and/or national research committee and with the 1964 Helsinki declaration and its later amendments or comparable ethical standards. All patients included in this study signed an informed consent to release their medical record data for research purposes. All patients were non-resective. The inclusion criteria were: age >18 years: HCC diagnosis based on the European Association for the Study of the Liver (EASL) Guidelines^15^; intermediate stage HCC (Barcelona Clinic Liver Cancer [BCLC] stage A or B); prior history of therapies for the disease; well compensated liver function classified as Child-Pugh Class A or B; absence of main trunk or segmental portal thrombosis; Eastern Cooperative Oncology Group (ECOG) performance status score of 0–2; and TACE treatment with CalliSpheres (CalliSpheres® Beads; Jiangsu Hengrui Medicine Co.Ltd., Jiangsu, China). Sixty patients with early and intermediate stage HCC referred to our department for TACE treatment October 2016 and December 2018 met the inclusion criteria and were included in the final analysis. These patients were treated with TACE using CalliSpheres of either 100–300 µm (small bead group) or 300–500 µm (medium bead group). Symptom control was assessed 3 months after completion of the TACE procedure.

### Imaging protocol

Abdominal and chest radiological imaging by computed tomography (CT; 64 slices, GE Revolution, USA) or magnetic resonance imaging (MRI; Ingenia 3 T, The Netherlands) was performed to assess hepatic tumor and extrahepatic disease status before TACE treatment (baseline). Follow-up imaging was scheduled 3 months after TACE treatment. For each patient, pre- and post-treatment CT/MRI images were independently reviewed by two board-certified radiologists, who were blinded to the treatment allocation. The largest tumor identified on the pre-treatment CT/MRI images was defined as the primary index tumor. This tumor was targeted during the TACE therapy and later assessed to determine the response to treatment.

### DEB-TACE with calliSpheres

The TACE procedure was performed by interventional radiologists in our department with >15 years’ experience with this approach. The transfemoral arterial access route was established using a 5 F vascular introducer (Terumo, Tokyo, Japan). Angiography of the celiac trunk and the superior mesenteric artery was acquired for characterizing the hepatic vascular anatomy using an angiography injection system (Mark V Provis, MERAD Inc., USA) and a 5 F catheter (COOK, IN, USA). In some patients, the angiography of abnormal extrahepatic branches was acknowledged based on careful review of the pre-TACE CT/MRI images or from the missing parts of the selective angiography showing pathological tumor vascularization.

Highly selective administration of the TACE treatment was performed. The pathological tumor-feeding vessels were selectively catheterized using a 2.7 F coaxial micro catheter (Progreat, Terumo, Tokyo, Japan). Super-selective arterial catheterization was performed whenever possible. When multiple arteries supplying the tumor were detected, transarterial embolization was performed with the catheter advanced into the arteries allowing for maximum perfusion.

DEB solution was prepared by mixing the CalliSpheres with 10 mg/10 mL doxorubicin (Jinyuan Pharmaceutical Manufacturing Co., Ltd., Changzhou, China) and diluting the suspension in 10 mL of 0.9% sodium chloride and 10 mL of iodinated contrast medium (omnipaque® = iohexol, GE Healthcare Company, UK). CalliSpheres with particle sizes of 100–150 µm were used in the initial 34 cases and CalliSpheres with particle sizes of 300–500 µm were used for the additional 26 cases. Patients in this study received a standard dose of doxorubicin at 75 mg/m^2^; in case of mild elevated bilirubin, the dose was reduced to 50 mg/m^2^ or 25 mg/m^2^. The DEB solution was injected slowly with a 5-mL syringe through the catheter under gentle pressure, until stasis or near-stasis was achieved in the tumor-feeding vessel^6^. A similar degree of selectivity was obtained for both groups. No antibiotic was given before or during the TACE procedure.

### Study endpoints

The primary endpoint was to evaluate complications and adverse events occurring within 3 months of the DEB-TACE using CalliSpheres, and the secondary endpoint was the tumor response rate. Adverse events were defined and graded according to the National Cancer Institute’s Common Terminology Criteria for Adverse Events version 4.0 (NCI-CTCAE v4). The modified Response Evaluation Criteria in Solid Tumors (mRECIST) was used to measure tumor response to the treatment. Measurements were expressed as mean ± standard deviation. Changes in tumor size and tumor contrast enhancement between pre- and post-TACE were expressed as percent change from baseline and categorized as complete response (CR), partial response (PR), stable disease (SD), and progressive disease (PD).

### Statistical analysis

The data were compared between the groups using the Mann-Whitney U test and Fisher’s exact test with SPSS software (v19.0, SPSS Inc., Chicago, IL, USA). P < 0.05 was considered statistically significant.

## Results

### Patients and treatment

The 60 patients in this study included 18 women and 42 men with an average age of 48 years (range, 35–72 years). The clinical characteristics of these patients at baseline are summarized in Table 1. In both groups, more than half of the cases were ECOG Performance Grade 0 (55%) and Child-Pugh Class A (58.3%), with no significant difference between the groups. Additionally, tumor size was similar between the groups at baseline.

**Table 1:** Patient characteristics at baseline.

Characteristics, n (%)	Small bead group (n = 34)	Medium bead group (n = 26)	P value
Diagnosis^#^			
Primary	18 (52.9)	14 (53.8)	0.342
Metastatic	16 (47.1)	12 (46.2)	0.251
Index tumor size (cm)	6.5 ± 1.2	5.8 ± 1.5	0.171
ECOG score			
0	19 (55.9)	14 (53.8)	0.371
1–2	15 (44.1)	12 (46.2)	0.228
Child-Pugh score			
A	20 (58.8)	15 (57.7)	0.294
B	14 (41.2)	11 (42.3)	0.183
Prior therapy for cancer			
Radiofrequency ablation	17 (50.0)	16 (61.5)	0.421
Systemic chemotherapy	17 (50.0)	10 (38.5)	0.203

^#^primary: HCC without intrahepatic metastasis.

metastatic: HCC with intrahepatic metastasis.

All patients received a doxorubicin dose up to 10 mg. No portal vein thrombosis or bile duct dilatation was observed at the time of the TACE procedure in any patient. The TACE procedure was performed without technical difficulties that would hinder the treatment of the index tumor.

### Tumor response

Response to treatment was evaluated at 3 months, and the results are shown in Table 2. The tumor response rate was classified according to the mRECIST criteria. The objective response rate (ORR, CR + PR) in patients receiving TACE with CalliSpheres of 100–300 µm (small bead group) was higher than that in patients receiving TACE with CalliSpheres of 300–500 µm (medium bead group; 64.8% vs. 56.2%), but the difference was not statistically significant (P > 0.05). The rates of SD were 7.6% in the small bead group and 20.8% in the medium bead group (P > 0.05), and the rates of PD were 17.6% in the small bead group and 23.0% in the medium bead group (P > 0.05). The majority of patients had 100% necrosis in both groups (76.5% in the small bead group and 73.1% in the medium bead group, P > 0.05), and 11.8% and 11.5% of patients in the small and medium bead groups, respectively, had a necrosis rate below 50% (P > 0.05).

**Table 2:** Tumor response at 3 months.

Clinical outcomes, n (%)	Small bead group (n = 34)	Medium bead group (n = 26)	P value
Tumor response by mRECIST			
CR	12 (35.4)	9 (33.1)	0.193
PR	10 (29.4)	6 (23.1)	0.153
SD	6 (7.6)	5 (20.8)	0.091
PD	6 (17.6)	6 (23.0)	0.241
Tumor necrosis			
100%	26 (76.5)	19 (73.1)	0.250
50–100%	4 (11.8)	4 (15.4)	0.371
<50%	4 (11.8)	3 (11.5)	0.290

### Adverse events and toxicity

**Figure 1:**
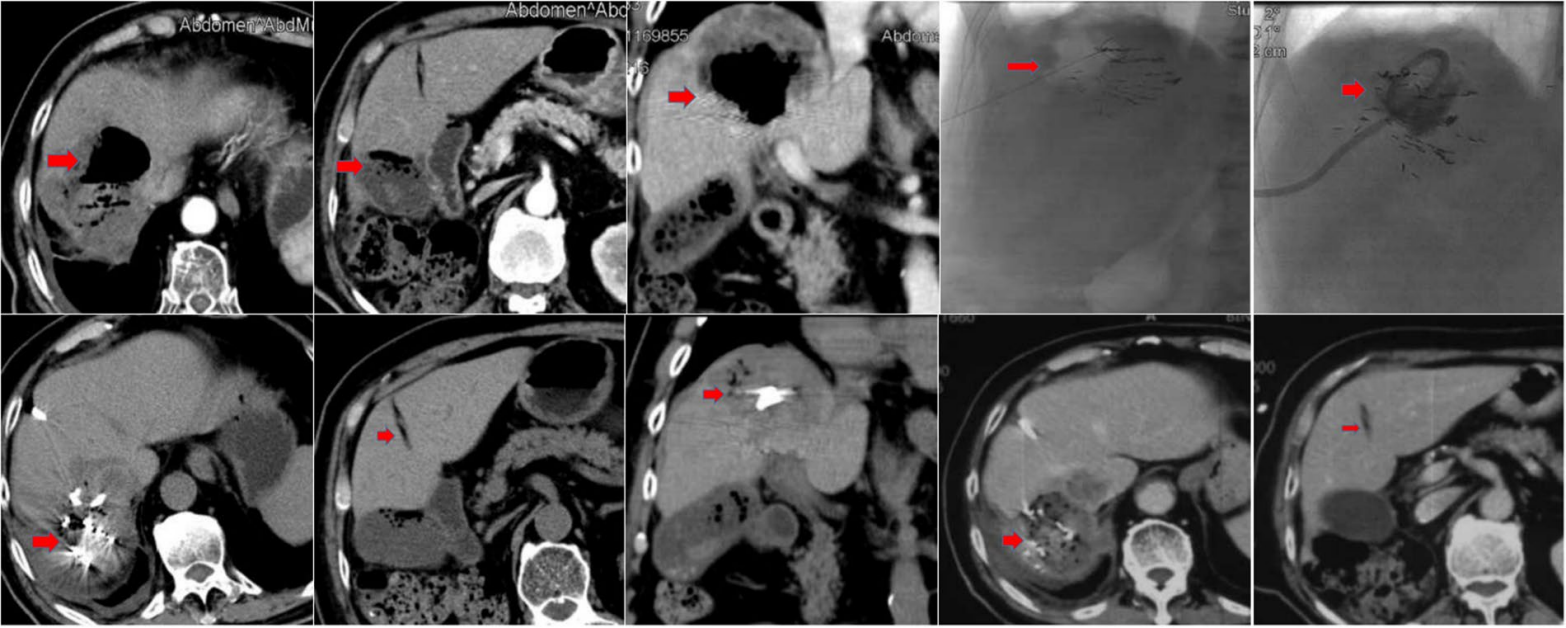
CT images of a 61-year-old male patient diagnosed with HCC. Four days after TACE (doxorubicin 50 mg and CalliSpheres of 100–300 µm, April 20, 2017), the patient suffered from an acute infection with biloma. The patient achieved a stable clinical condition after external drainage and antibiotic therapy. The top left CT images show biloma formation (April 24, 2017). The top right CT images show external drainage of biloma (April 24, 2017). The bottom left CT images show complete disappearance of the biloma 1 day after treatment (April 25, 207). The bottom right CT images show tumor necrosis with no biloma (July 4, 2017). The patient was in stable condition.

**Figure 2:**
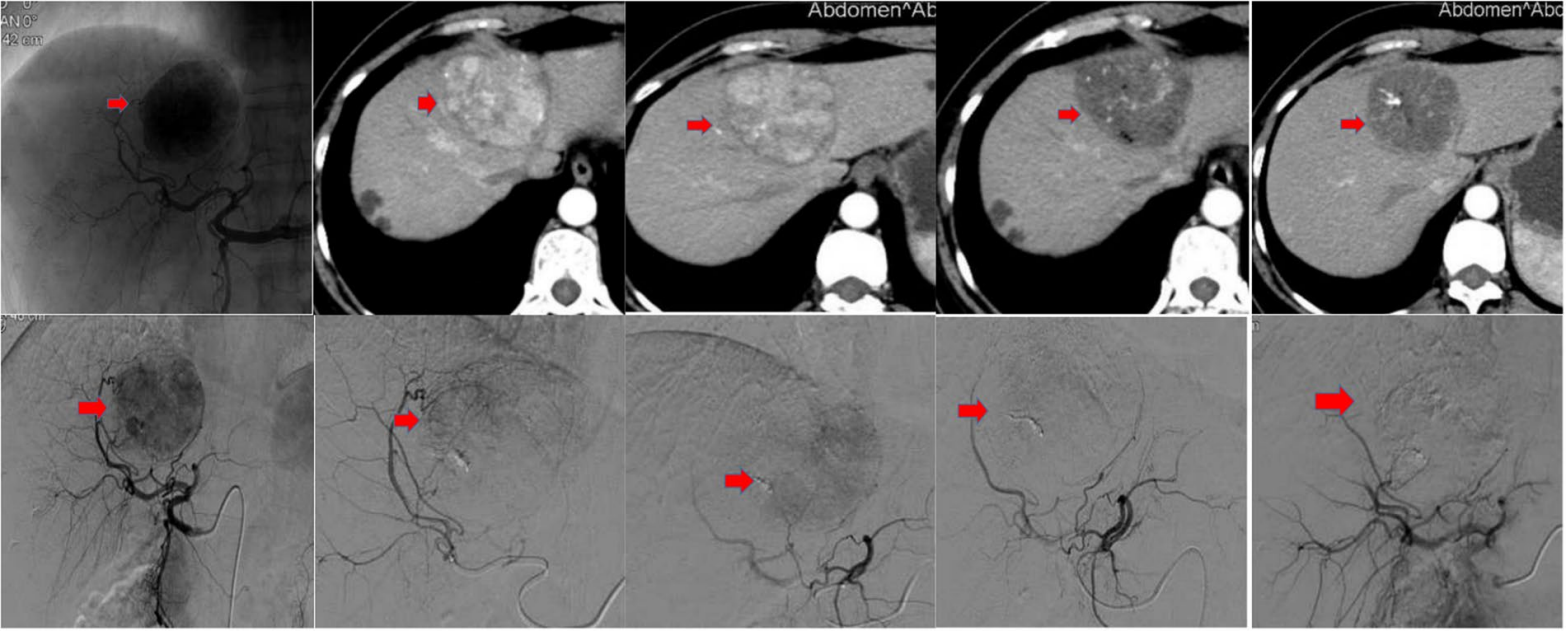
CT images of a 53-year-old female patient diagnosed with HCC. The Top left CT image shows a large tumor in the left liver lobe (May 18, 2017). The top middle images show residual enhancement of the tumor in the left liver lobe (June 20, 2017). The bottom CT images show TACE with doxorubicin 50 mg and CalliSpheres of 300–500 µm (June 22, 2017). The patient reported fever and abdominal pain after the TACE procedure, but no biloma was detected (top right, June 26, 2017).

All patients were monitored for toxicity. No deaths related to the TACE procedure occurred. The adverse events observed within 3 months of the procedure are summarized in Table 3. Grade 3/4 adverse events were noted in 8 (23.5%, 8/34) patients, all of whom belonged to the first 34 patients who received TACE with CalliSpheres of 100–300 µm (small bead group). The grade 3/4 severe adverse events included ischemic hepatitis (23.5%, 8/34), liver abscess (5.9%, 2/34), and biloma (5.9%, 2/34) (Fig. 1). Emergency CT/MRI scan and drainage were performed in these patients, and no cases of post-procedure mortality occurred in 30 days. In the other 26 patients who received TACE with CalliSpheres of 300–500 µm, no severe complications were noted (Fig. 2). No systemic adverse events related to nontarget embolization, including pulmonary embolism, splenic infarction, spinal cord injury, and acute pancreatitis or cholecystitis, were recorded^16^. The median hospital stay was 5 days for the small bead group (range, 2–7 days) and 4 days for the medium bead group (range, 2–6 days; P > 0.05). Post-embolization syndrome (PES) occurred 26.5% (9/34) of patients in the small bead group and 23.1% (6/26) of patients in the medium bead group (P > 0.05). The most frequent adverse events were abdominal pain (52.9%, 18/34 in the small bead group vs. 38.5%, 10/26 in the medium bead group; P > 0.05) and nausea/vomiting (26.5%, 9/34 in the small bead group vs. 38.5%, 10/26 in the medium bead group; P > 0.05), which were treated with analgesic drugs and anti-emetics, respectively, followed by mild ascites (8.8%, 3/34 in the small bead group vs. 3.8%, 1/26 in the medium bead group; P > 0.05). In addition, transient post-procedure grade 1–2 elevation of transaminase and bilirubin levels was found in 76.5% (26/34) in the small bead group and 57.7% (15/26) in the medium bead group (P > 0.05).

**Table 3:** Adverse events.

Toxicity (CTCAE v4), n(%)	Small bead group (n = 34)	Medium bead group (n = 26)	P value
Ischemic hepatitis	8 (23.5)	0 (0)	0.003
Liver abscess	2 (5.9)	0 (0)	0.016
Biloma	2 (5.9)	0 (0)	0.022
Grade 3/4 toxicity	8 (23.5)	0 (0)	0.009
Post-embolization syndrome	9 (26.5)	6 (23.1)	0.061
Abdominal pain	18 (52.9)	10 (38.5)	0.153
Nausea/vomiting	9 (26.5)	10 (38.5)	0.281
Mild ascites	3 (8.8)	1 (3.8)	0.305
Grade1/2 elevation of transaminase and bilirubin	26 (76.5)	15 (57.7)	0.177

## Discussion

TACE is considered the standard treatment for unresectable HCC^17^, but variability in TACE techniques has been shown to affect patient outcomes across hospitals and countries. TACE with doxorubicin-loaded DEBs has the potential to standardize techniques and improve clinical benefits in patients with HCC, with some clinical trials showing promising results in terms of tumor response and safety. However, most studies have been carried out in a limited number of patients or have focused on specific groups of patients, such as patients with early stage tumors. DEB-TACE was effective and tolerable in Chinese HCC patients with portal vein invasion, and previous C-TACE treatment appeared to be an important predictor of worse clinical outcome^18^. Moreover, the optimal particle size of DEBs for HCC patients receiving DEB-TACE treatment remains uncertain and not well studied^11,19–22^. Lencioni *et al*.^23^ recommended that, in order to embolize the tumor thoroughly, a particle size range of 300–500 um is needed for tumors >5 cm. For tumors <3 cm, particle size ranges of 75–150 µm or 100–300 µm were selected according to the tumor blood supply. For tumor with rich blood supply, 100–300 µm was the first choice, and for tumors with super abundant blood supply, 300–500 µm was the first choice. For tumors with poor blood supply, 100–300 µm was considered as the most appropriate size. A previous study assessed the efficacy and safety of DEB-TACE with different particle sizes and found that doxorubicin-loaded DEBs of small size were favorable over those of medium size for HCC treatment^24^, but few studies have been carried out in patients who have already tried several rounds of treatment for liver cancer. To our best knowledge, this is the first retrospective comparative study of the clinical benefit and safety of CalliSpheres of different particle sizes for patients who had received multiple rounds of conventional treatment for liver cancer.

In this study, we compared the clinical responses of patients treated with TACE with doxorubicin-loaded DEBs of particle sizes 100–300 µm or 300–500 µm. This comparison is of clinical significance because there is widespread concern over use of DEBs of particles smaller than 300 µm for DEB-TACE due to PES. In our study, patients treated with CalliSpheres of 300–500 µm achieved an ORR of 56.2%. This is comparable to the results of the first major prospective clinical trial of DEB-TACE for HCC, in which doxorubicin-loaded DEBs with particle sizes of 300–500 µm were used and an ORR of 52% was achieved based on the imaging criteria of EASL^4^. In this study, no major local or systemic complications, including PES, were identified in patients treated with DCB-TACE using beads of 300–500 µm, demonstrating that this is a safe and effective technique for patients with liver tumors who have undergone several rounds of oncology therapies before TACE. CalliSpheres of 100–300 µm resulted in a higher ORR (64.8%) according to mRECIST without damage to the adjacent healthy liver, but the incidence of severe adverse events in patients with liver tumors was higher than that after treatment with medium-sized beads. In this study, toxicity related to TACE with CalliSpheres for liver tumors was mainly limited to PES, which was observed in 25% of patients, all of whom were in the small CalliSphere group. There were two cases of post-embolization biloma, which were treated by immediate percutaneous drainage. No case with post-embolization liver failure was observed. It is known that patients with biliodigestive anastomosis or biliary stents *in situ* have a higher risk of developing post-chemoembolization liver abscess, regardless of the prophylactic administration of broad-spectrum antibiotics^25^. Although no patient reported a previous history of bile duct surgery, all patients in this study had undergone several rounds of intervention therapies, including systemic chemotherapy, C-TACE, and radiofrequency ablation (RFA). Some previous studies reported post-embolization liver necrosis and intrahepatic biloma formation occurring in approximately half of patients treated with doxorubicin-loaded DEBs of small particle size (100–300 µm), but such events were rare in patients receiving ethiodized oil-based C-TACE^26,27^. This is in agreement with our finding that PES occurred more frequently in patients treated with small CalliSpheres compared with medium-sized CalliSpheres. The CalliSphere beads used in this study were made of polyvinyl alcohol. Loading of the chemotherapeutic agent causes the caliber of the CalliSpheres to shrink by 50%, making it easier for them penetrate into more distal tumor arterioles. Upon release of the loaded medicine in the distal arterioles, the beads return to their original size and consequently obstruct the microarteries^14^. Another possible explanation for PES related to the small size DEBs is that the smaller size makes the DEBs more easily penetrate the normal residual liver parenchyma more deeply, which consequently induces irreversible ischemia in the liver and biliary system^26^. This hypothesis was supported by the observation that, in patients receiving DEB-TACE treatment, those with a lower hepatic tumor burden experienced more bilomas than those with a higher tumor burden, indicating high sensitivity of the residual normal liver parenchyma to ischemic changes after treatment with DEBs of small size^26^. In our study, CalliSpheres of 100–300 µm and 300–500 µm in their original state shrank to 50–150 µm and 150–250 µm, respectively, after loading with doxorubicin, and returned their original size after releasing doxorubicin at the target tissue. This mechanism involving a shrinkage-recovery process is favorable for a more distal embolization during TACE treatment for liver tumors but might be create a higher risk of necrotic complications.

This study has several limitations. First, the retrospective nature of this study involved potential selection bias that could lead to inaccurate results. Specifically, instead of randomized allocation, the first 34 patients and then the remaining 26 patients received TACE treatment with CalliSpheres of 100–300 µm and 300–500 µm, respectively. Although no obvious differences in patient characteristics were observed between the groups and no other changes were made to the TACE technique during the study period, possible bias resulting from the time difference cannot be excluded. Second, because the ORR was determined based solely on the index tumor, the true tumor response rate might be different if secondary or smaller tumors were included in the analysis. However, incorporating more tumors into the assessment could make the analysis more complicated and the results more difficult to interpret. Response rate calculation based on the index tumor is widely applied in research and has been shown to be a good surrogate for survival in HCC^28^. Third, the relatively small sample size may reduce the statistical power to detect a difference between patients treated with CalliSpheres of different sizes. In addition, because the patient population in this study was heterogeneous in terms of tumor diagnosis and previous treatment regimens, the findings observed in these patients may not be generalizable to the general liver cancer patient population. Fourth, long-term overall survival could not be analyzed due to the short follow-up period. Prolonged overall survival is the gold standard measure for treatment efficacy, although some evidence supports an association between radiographic response and overall survival^29^. Finally, this study only compared CalliSpheres of small and medium sizes. The clinical performance of CalliSpheres of other particle sizes remains to be discovered.

In conclusion, our retrospective study demonstrated a comparable tumor response rate but lower frequencies of PES and other complications following DEB-TACE using CalliSpheres of 300–500 µm in patients with HCC, compared with treatment using smaller CalliSpheres, providing evidence that DEB-TACE with CalliSpheres of 300–500 µm is an effective and safe treatment choice for patients with intermediate stage liver cancer who have undergone various oncological treatment regimens. Further prospective randomized studies with larger numbers of patients, a longer follow-up period, ideal allocation to treatment groups with different sizes of CalliSpheres, and baseline stratification according tumor characteristics are needed to identify patients who are most likely to respond to DEB-TACE.

### Research involving human participants and/or animals

In this retrospective study, medical records of selected patients were reviewed. This study was approved by the Ethics Committee of our hospital, and all patients included in this study signed an informed consent to release their medical record data for research purposes.

### Informed consent

All patients included in this study signed an informed consent to release their medical record data for research purposes.
